# Time course profiling of the retinal transcriptome after optic nerve transection and optic nerve crush

**Published:** 2008-06-03

**Authors:** Marta Agudo, Maria Cruz Pérez-Marín, Ulrika Lönngren, Paloma Sobrado, Ana Conesa, Isabel Cánovas, Manuel Salinas-Navarro, Jaime Miralles-Imperial, Finn Hallböök, Manuel Vidal-Sanz

**Affiliations:** 1 Departamento Oftalmología, Facultad de Medicina, Universidad de Murcia, Murcia Spain; 2 Hospital Universitario Virgen de la Arrixaca, Murcia, Spain; 3 Department of Neuroscience, Unit for Developmental Neuroscience, Uppsala University, Uppsala, Sweden; 4 Bioinformatics Department Centro de Investigación Príncipe Felipe, Valencia, Spain

## Abstract

**Purpose:**

A time-course analysis of gene regulation in the adult rat retina after intraorbital nerve crush (IONC) and intraorbital nerve transection (IONT).

**Methods:**

RNA was extracted from adult rat retinas undergoing either IONT or IONC at increasing times post-lesion. Affymetrix RAE230.2 arrays were hybridized and analyzed. Statistically regulated genes were annotated and functionally clustered. Arrays were validated by means of quantative reverse transcription polymerase chain reaction (qRT–PCR) on ten regulated genes at two times post-lesion. Western blotting and immunohistofluorescence for four pro-apoptotic proteins were performed on naïve and injured retinas. Finally, custom signaling maps for IONT- and IONC-induced death response were generated (MetaCore, Genego Inc.).

**Results:**

Here we show that over time, 3,219 sequences were regulated after IONT and 1,996 after IONC. Out of the total of regulated sequences, 1,078 were commonly regulated by both injuries. Interestingly, while IONT mainly triggers a gene upregulation-sustained over time, IONC causes a transitory downregulation. Functional clustering identified the regulation of high interest biologic processes, most importantly cell death wherein apoptosis was the most significant cluster. Ten death-related genes upregulated by both injuries were used for array validation by means of qRT–PCR. In addition, western blotting and immunohistofluorescence of total and active Caspase 3 (Casp3), tumor necrosis factor receptor type 1 associated death domain (TRADD), tumor necrosis factor receptor superfamily member 1a (TNFR1a), and c-fos were performed to confirm their protein regulation and expression pattern in naïve and injured retinas. These analyses demonstrated that for these genes, protein regulation followed transcriptional regulation and that these pro-apoptotic proteins were expressed by retinal ganglion cells (RGCs). MetaCore-based death-signaling maps show that several apoptotic cascades were regulated in the retina following optic nerve injury and highlight the similarities and differences between IONT and IONC in cell death profiling.

**Conclusions:**

This comprehensive time course retinal transcriptome study comparing IONT and IONC lesions provides a unique valuable tool to understand the molecular mechanisms underlying optic nerve injury and to design neuroprotective protocols.

## Introduction

Optic nerve injury triggers retinal ganglion cell (RGC) death [1]. The progression of this death depends on the type of lesion, crush or transection, and on its distance from the eye [2-5].

The effects of intraorbital nerve transection (IONT) and intraorbital nerve crush (IONC) on retinal degeneration, and more specifically on RGC death, have been thoroughly studied by our group [5-8]. Both injuries trigger a massive RGC loss, which is slower and less acute after crush than after transection [3,4]. Thus, anatomically, it is observed that 38% and 20% of RGCs are lost seven days post-optic nerve transection or crush, respectively. Our IONT injury cleanly severs all the RGC axons, sparing the blood supply. The IONC injury compresses the blood vessels and axons in 10 s. Axons are thus severed by the acutely exerted pressure and by the glial reaction that occurs later in the retina and around the lesion site [1,9-13]. During the crush, the retinal blood supply is transiently hampered but in the present study we did check that our surgical manipulation avoided an ischemic insult.

Numerous efforts have been made to slow down RGC death triggered by optic nerve injury. So far none of them have successfully delayed RGC death beyond 15 days post-optic nerve injury [6-8,14-19]. Therefore, we purposed to investigate the molecular signals triggered by optic nerve injury in the retina. We have performed an exhaustive time-course analysis of the retinal transcriptome profile comparing mRNA expression from IONC and IONT retinas to naïve retinas using Affymetrix RAE230.2 arrays.

Sequences significantly regulated after IONT and after IONC at each time point were extracted and analyzed. Data were compared to learn the differences and similarities between both injuries. An extensive functional clustering was done, revealing that optic nerve injury alters highly important biologic functions in the retina as cell death, visual perception, and cytoskeleton. Due to the large number of regulated sequences and because RGC death is the most demolishing effect of optic nerve injury, we focused this work on the study of regulated genes involved in this process.

## Methods

### Animal handling and surgery

One hundred and forty adult female Sprague-Dawley rats (180–220 g bodyweight) were used for the array study and 48 for western and immunohistofluorescence studies. Rats were obtained from the university breeding colony. For anesthesia, we used xylazine (10 mg/kg bodyweight; Rompun; Bayer, Kiel, Germany) and ketamine (60 mg/kg bodyweight; Ketolar; Pfizer, Alcobendas, Madrid, Spain). All experimental procedures were performed in accordance with the Association for Research in Vision and Ophthalmology guidelines for the use of animals in research. Sterile precautions were maintained for all surgical procedures.

**Table 1:** Number of pooled biologic array replicas per lesion and time point.

Time post lesion							
Experimental Group	No lesion	12 h	24 h	48 h	3 d	7 d	15 d
Control	5						
IONC		3	3	3	3	3	
IONT		3	2	3	2	3	2

This table shows the number of arrays hybridized per lesion and time point under analysis. To avoid technical variability several arrays replicas were performed for each lesion and time point. To avoid biologic variability, each replica was hybridized with an RNA pool of 4 retinas from different animals. RNA extraction for each replica was performed separately as follows: naïve replica 1 was hybridized with RNA extracted from retinas 1 to 4; naïve replica 2 was hybridized with RNA extracted from retinas 5 to 9 and so on. Abbreviations: IONT: intraorbital nerve transection, IONC: intraorbital nerve crush,, h: hours, and d: days.

#### The array experiment

Animals were divided into a control group (naïve), which did not undergo any experimental manipulation (n=20) and two experimental groups, one receiving an intraorbital nerve transection (IONT, n=60) and the other an intraorbital nerve crush (IONC, n=60). The left optic nerve was intraorbitally axotomized according to procedures that are standard in our laboratory [3-5,11]. Briefly, an incision was made in the superior orbital rim, the superoexternal orbital contents were dissected, and the superior and external rectus muscles were removed. When performing IONT, the dura mater was opened longitudinally to spare the blood supply, and the optic nerve was transected 0.5 mm from the optic disc [8,14]. The purpose of the IONC injury was to crush the entire population of optic nerve fibers. This lesion was performed by crushing the optic nerve 3 mm from the optic disc for 10 s with a pair of watchmaker’s forceps. Before and after the procedure, the eye fundus was observed through the operating microscope to assess the integrity of the retinal blood flow. In the very few instances in which blood supply did not restore within the following minutes, the animal was discarded.

#### Western blotting analyses

Animals were subjected to surgery as above (IONT and IONC n=4 per time point and lesion).

#### Immunohistofluorescence analyses

To identify RGCs in the sectioned retinas, they were retrogradely traced by applying Fluorogold (3% diluted in 10% DMSO-saline, Fluorochrome, LLC, Denver, CO) to the superior colliculus (SC) one week before the optic nerve injury was performed, as previously described [11-13]. Briefly the rats were anesthetized, each midbrain was exposed and, after removing the piamater overlying the SC, a small piece of gelatine sponge (Spongostan Film, Ferronsan, Denmark) soaked in 3% FG, diluted as aforementioned, was laid over the SC to label RGCs by retrograde axonal transport. Three animal groups were made: naïve, IONT, and IONC (n=5 per group). Optic nerve injury was performed as aforementioned, and animals were processed 48 h post lesion (hpl).

### Array experimental procedures

#### Tissue processing and RNA extraction

Animals were kept the appropriate time post-injury (12 h, 24 h, 48 h, 3 days, 7 days, and 15 days, n=8–12 per time point) and then sacrificed by an intraperitoneal overdose of sodium pentobarbital. Left retinas were freshly dissected and immediately frozen in liquid nitrogen. Four retinas from each time point, animal group, and biologic replica were pooled, and RNA was extracted using Trizol (Invitrogen, Barcelona, Spain). RNA was further cleaned through RNeasy mini kit columns (Qiagen, Izasa, Madrid, Spain). The RNA integrity was checked using a Bioanalyzer (Agilent Technologies, Santa Clara, CA), and concentrations were determined using a NanoDrop (NanoDrop Technologies, Wilmington, DE).

#### Array experiment

RNA was labeled, arrays were hybridized, and a signal was acquired at the Genomic Service (Universidad Complutense de Madrid, Madrid, Spain) as described in [20]. Briefly, cDNA, which was created from 5 μg of each RNA sample, was synthesized with One-Cycle cDNA synthesis kit of Affymetrix (Santa Clara, CA) according to the Gene Chip Expression Analysis Technical Manual (2003; Affymetrix). From this cDNA, cRNA was synthesized according to the protocol of IVT Labeling kit of Affymetrix. Finally, this cRNA was purified with the Gene Chip Sample Cleanup Module of Affymetrix, recovering it in 22 μl of water. cRNA (15 μg) was fragmented to prepare the hybridization cocktails. Samples were hybridized to Rat genome 230 arrays (rat RAE230.2, Affymetrix), which are two gene chips that contain 31,000 probe-sets (genes or expressed sequence tags [ESTs]). Following hybridization, the gene chips were washed, stained, scanned, and analyzed with Gene Chip Operating Software (GCOS 1. 2; Affymetrix). The quality of the experiments was controlled by the visual inspection of the gene chips and by the presence of the spike controls and housekeeping control genes. A total of 35 gene chips were analyzed.

Each array replica was hybridized with an RNA pool of four retinas from independent RNA extractions and different animals (i.e., Array 1 was hybridized with naïve RNA from animals 1–4, array 2 with naïve RNA from animals 5–8 and so on). See Table 1 for a summary.

#### Array normalization and analysis

Expression data in the CEL file format were imported directly into Bioconductor software for normalization and analysis [21]. Normalization was performed using the package justGCRMA (Robust Multi-Array expression measure using sequence information). To check the linearity and reproducibility that represent an adequate normalization of the arrays, scatter plots were performed between the log_2_ intensities of the injured retina arrays and the naïve retina arrays. Raw and normalized data for all hybridizations have been deposited as data series in NCBIs Gene Expression Omnibus (GEO, and are accessible through GEO Series accession number GSE9918).

The results from the IONT- and IONC-array analyses were compared to the results from the naïve-array ones. This allowed the identification of IONT- and IONC-regulated sequences. Statistically significant regulated sequences at each time point were extracted using the Limma package (Data analysis, linear models, and differential expression for micro array data; bioinf) with a cut-off for those sequences with a B value >0 and an adjusted p value false discovery rate (FDR) <0.01 and with a minimum regulation of 20% when compared to control. However, sequences modestly regulated along time would be missed with the Limma analysis since they would not be considered significant. To avoid this bias we also performed an analysis of the temporal regulation using the maSigPro package [21]. This package is specifically designed to analyze and compare time course experiments (p value FDR less than 0.01 and R^2^ threshold equal to 0.6). Log_2_ output values of regulated sequences were transformed to their net values (fold change). It is important to highlight here that when a sequence is not expressed, its mRNA level is 0. For this reason, the control level of expression was set to 1, any value less than 1 and more than 0 will then indicate downregulation and everything above 1 will indicate upregulation. IONT- and IONC-regulated sequences (p value <0,01, B value >0) were annotated and functionally clustered using the Database for Annotation, Visualization and Integrated Discovery, (DAVID [22]), the Kyoto Encyclopedia of Genes and Genomes database (KEGG), and PubMed reports. Clustering (molecular function and biologic process) was done using both Affymetrix RAE230.2 and the *Rattus norvegicus* genome as the background. Clusters that were significantly regulated were detected using the EASE tool [23] (p value FDR <1.00E-04). Finally, all regulated genes were imported to MetaCore^TM^ software (GeneGoLtd .) to generate “signaling maps” that include molecular interactions between the regulated sequences that can be associated to certain biologic processes such as cell death. Significantly regulated Metacore^TM^ cell-death signaling maps (pEASE <1.00E-04) together with their corresponding curated interactions were merged, and custom maps for IONT and IONC gene regulation were generated using the MapEditor^TM^ software (GeneGo Ltd).

### Quantitative real time polymerase chain reaction

One microgram of total RNA was used for cDNA synthesis using random hexamer primers (TaqMan Reverse Transcriptase, Applied Biosystems, Foster City, CA). Analysis of mRNA levels was performed using iQ SYBR Green Supermix (Bio-Rad Laboratories, Hercules, CA) in combination with a MyiQ Single Color Real-Time PCR Detection System (Bio-Rad Laboratories) and sequence specific primers (Table 2). Primers were designed using the software Primer Express 2.0 (Applied Biosystems) to target the same regions as the Affymetrix probes (predominantly in the 3′−UTR region). Melting temperature for the primers were approximately 59 °C and amplicon length approximately 80 bp. Primer concentrations were optimized for each primer pair and ranged between 150 and 500 nM final concentration. Polymerase chain reactions (PCRs) were performed in triplicate in a 96 well plate according to the manufacturer’s protocol. For each series, a threshold cycle value (C_t_) corresponding to the PCR cycle at which the fluorescent emission reaches a threshold above the baseline emission was determined. Based on this, initial mRNA levels were calculated. To confirm specificity of amplified PCR products, dissociation curve analyses and agarose gel electrophoresis were performed. As control genes *RPL30, RPL31, RPL37, β-actin*, and *hypoxanthine guanine phosphoribosyl transferase (HPRT)* were measured. These control genes were chosen because no change among samples was observed in the array experiment. Initial mRNA levels were normalized to the average mRNA levels of *RPL30, RPL31*, and *RPL37* to correct for variations in the cDNA synthesis. Relative mRNA levels in naïve retinas were used as a reference.

**Table 2:** Primers used for qRT–PCR array validation

Target genes^a^	Sequence of forward and reverse primer^b^
Ahr NM013149	CCAAAACAGATCTCAATGCTGGT (2670–2692), TCAGAGCATCCCACCTTATAGGTAA (2750–2726)
Calr NM022399	CAGGTATGGAGGAAAAGCCCTAG (1597–1619), TCCTACCCTCCTCTGAGATTCAGA (1668–1645)
Ccl2 NM031530	CAACCCTAAGGACTTCAGCACCT (395–417), TGTGGAAAAGAGAGTGGATGCAT (474–452)
Clu NM053021	GGTCTCCACAGTGACAACCCA (1149–1169), TCAAACAGCTTCACCACCACC (1226–1206)
Eef2k NM012947	TGGTGACAGGCAGTCCATGAT (2107–2127), CTTCCGACCAGTCTTGACACCT (2195–2174)
Lcn2 NM130741	CCTGTACGGAAGAACCAAGGG (575–595), CCAGAGACTTGGCAAAGCTGA (644–625)
Litaf XM343856	CTGTGGTACGGTCATCATGGTCT (1646–1668), GCCCTGAACTTATCTTTGTCGTTG (1722–1699)
Stat1 NM032612	TTGACAAAGACCATGCCTTCG (2300–2320), TCATCGAGCTCCATCGGTTC (2372–2353)
Tnfrsf1a NM013091	GCCAGGAGAGGTGATTGTGG (1880–1899), CCCTGAGAAGCTTTGTTTGGG (1963–1943)
Tnfsrsf12a NM181086	AACACTGGGTTCCACCCACTC (741–761), TCTAACCCACTCCCAGGGCTA (813–793)
β*-*actin NM031144	CTTCAACACCCCAGCCATG (456–474), GTGGTACGACCAGAGGCATACA (524–503)
Hprt NM012583	CTTGCTCGAGATGTCATGAAGG (211–232), GCAAAGAACTTATAGCCCCCCT (293–272)
RPL30 NM022699	CAACTGTCCAGCTTTGAGGAAA (223–244), TGATGGACACCAGTTTTAGCCA (291–270)
RPL31 NM022506	CATCAACGAGGTGGTGACCC (225–244), TCTTGAAGCCCACTCCATGG (295–276)
RPL37 NM031106	TCCAAGGCCTACCACCTTCA (70–89), CTTTCTCTTGCGCTTGGCA (138–120)

Information in this table shows in ^a^Gene symbol and GenBank accession number and in ^b^numbers indicating position of the primers in the cDNA.

### Immunohistofluorescence

Naïve, IONT-, and IONC-injured animals whose retinal ganglion cells had been previously traced with Fluorogold (n=5 per group, 48 h post-axotomy) were deeply anesthetized with pentobarbital and perfused transcardially with 4% paraformaldehyde (PFA in 0.1 M phosphate buffer) after a saline rinse. Retinas were dissected and post-fixed for 2 h at 4 °C before switching to 30% sucrose (Sigma, Alcobendas, Madrid, Spain) for at least 12 h. Tissue was embedded in optimal cutting temperature (OCT) compound (Sakura Finetek, Torrance, CA), frozen on liquid nitrogen, and kept at −70 °C. Retinas were sectioned at a thickness of 15 μm on a cryostat. Sections were blocked in 2% donkey serum in phosphate buffered saline (PBS) with 0.1% Triton (PBST) and incubated overnight a 4 °C with the appropriate antibody. Immunoreactivity was detected using fluorescence-conjugated secondary antibodies (4 h at room temperature in PBST). Images were taken with a CCD camera using the ImageProPlus software (Image-Pro® Plus 5.1 for Windows®; Media Cybernetics, Inc., Silver Spring, MD) and further processed with Adobe Photoshop 7.0 (Adobe Systems Inc., San Jose, CA).

### Western blot analysis

Freshly dissected retinas from naïve, IONT-, and IONC-injured animals (12 h, 48 h, and 3 days or 7 days post-axotomy, n=4 per group and time point) were immediately frozen in liquid nitrogen and stored at −70 °C until protein was extracted. Tissue was homogenized in 300 μl of lysis buffer (1% Nonidet-p40, 20mM Hepes pH 7.4, 100 mM NaCl, 100 mM NaF, 1 mM Na_3_VO_4_, and 5 mM EDTA with 1X protease inhibitor cocktail, Roche Diagnosis, Barcelona, Spain) and then incubated on ice for 2 h. After centrifugation at 18928x g for 15 min at 4 °C, the supernatant was collected to establish total protein values. The amount of protein was determined using a bicinchoninic acid (BCA) protein assay kit (Pierce, ThermoFisher Scientific, Cultek SL, Madrid, Spain) and β-actin detection. β-actin western blots were scanned, and intensities were measured (Gene Tool, Syngene, Synoptics Ltd, Cambridge, UK). If necessary, the loading volume was corrected to equal the amount of loaded protein among all samples. Protein (20–60 μg) was loaded on 16% or 12% SDS-polyacrylamide gels for standard electrophoresis. Proteins were transferred to a polyvinylidene fluoride (PVDF; Amersham, GE Healthcare Europe GmBH, Barcelona, Spain) membrane (Amersham Pharmacia Biotech). Membranes were blocked with 5% fat free milk in PBS with 0.1% Tween 20 and incubated with the appropriate antibody overnight at 4 °C. Membranes were then incubated with horseradish peroxidase (HRP)-conjugated secondary antibodies. HRP activity was visualized by applying chemiluminescent substrate (Enhanced Chemo Luminiscence (ECL), Amersham, GE Healthcare Europe GmBH, Barcelona, Spain) followed by exposure of the membrane to an X-ray film. The exposed films were analyzed with the Gen Tools program (Syngene). The signal intensity of treated retinas was referred to the signal in naïve ones, which were arbitrarily considered as 100%. To avoid biologic variability, extracts from four animals were loaded in parallel, and to avoid technical variability, each western analysis was performed three times. Data shown are the averaged values of these replicas with their standard error of the mean (SEM) value.

**Figure 1:**
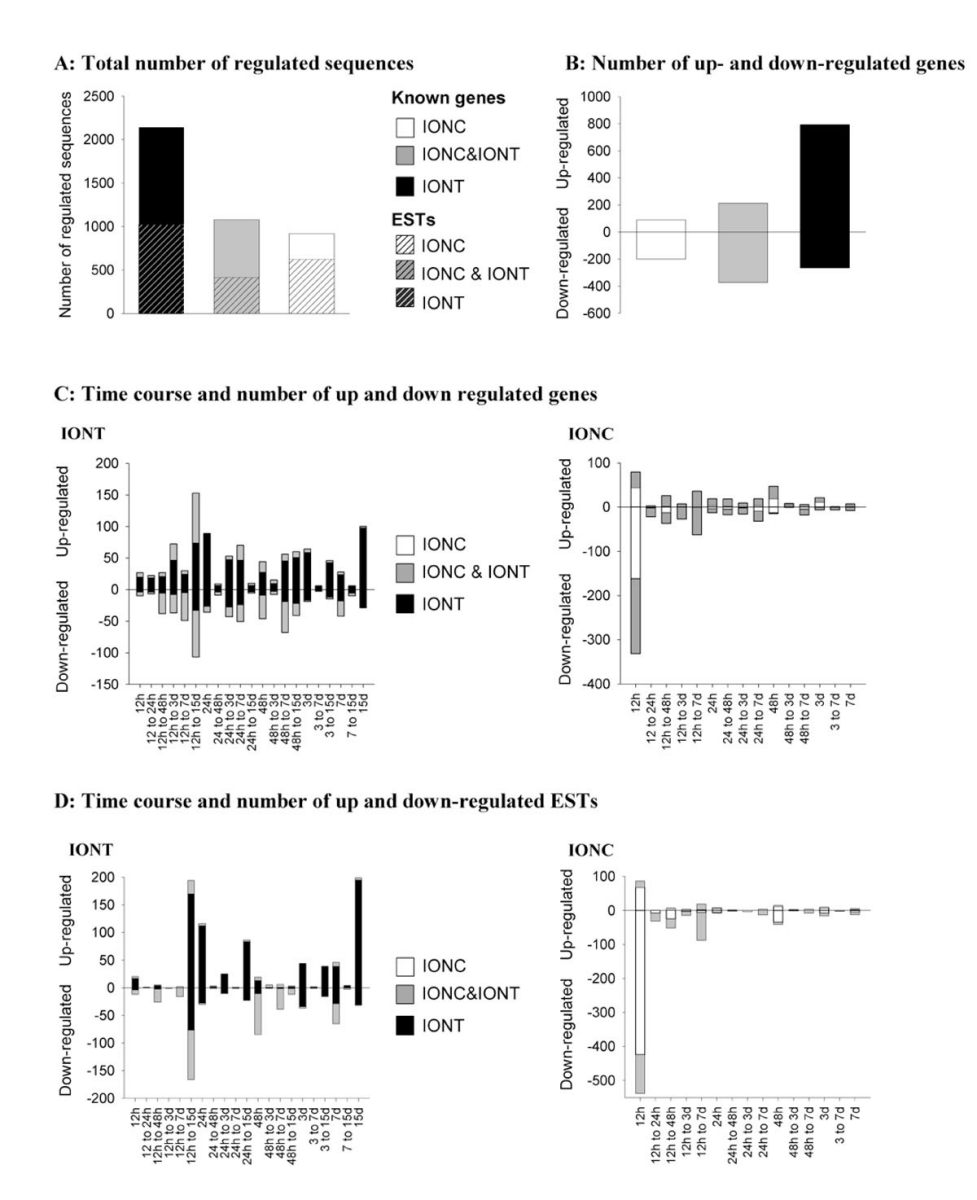
Time course and number of sequences regulated after optic nerve injury in the adult rat retina. **A:** Graph depicting the total number of sequences regulated in the retina specifically by intraorbital nerve transection (IONT; black columns), specifically by intraorbital nerve crush (IONC; white columns) and commonly by both lesions (gray columns) **B:** In this graph is shown the number of genes specifically up or down-regulated in the retina by IONT (black columns), specifically up or down-regulated by IONC (white columns) and commonly up or down-regulated by both lesions (gray columns). **C:** Graph on the left illustrates the time course and number of genes up or down-regulated in the retina at each time after IONT (black bars represent genes specifically regulated by transection and gray bars genes regulated by transection and crush). Graph on the right illustrates the time course and number of genes up or down-regulated in the retina at each time after IONC (white bars represent genes specifically regulated by crush and gray bars genes regulated by transection and crush). **D:** Graph on the left illustrates time course and number of expressed sequence tags (ESTs) up or down-regulated in the retina at each time after IONT (black bars represent ESTs specifically regulated by transection and gray bars ESTs regulated by transection and crush). Graph on the right illustrates the time course and number of ESTs up or down-regulated in the retina at each time after IONC (white bars represent ESTs specifically regulated by crush and gray bars ESTs regulated by transection and crush). In **C** and **D** the regulated sequences (either genes or ESTs) were clustered according to when they started to show regulation and how long they were regulated either at one time point (12 h, 24 h, etc) or at several (regulated from 12 h to 24 h, from 12 h to day 3, etc). The number of upregulated sequences is shown in the positive y-axis and number of downregulated ones in the negative y-axis. All sequences were significantly regulated (p value FDR <0,01 and a B value >0) compared to naïve expression levels.

### Antibodies and working dilutions

All antibodies were diluted in phosphate buffered saline with 0.1% Triton (PBSTx) or phosphate buffered saline with 0.1% Tween 20 (PBSTw) for immunohistofluorescence and western blotting experiments, respectively.

#### Primary antibodies

Rabbit anti-β-actin (Santa Cruz Biotechnologies, Heidelberg, Germany) was diluted at 1:200 for western blotting analysis. Rabbit anti-TRADD (Abcam, Cambridge, UK) was diluted 1:70 for western blotting and 1:50 for immnunofluorescence experiments. Mouse anti-TNF- R1 (H-5, Santa Cruz Biotechnologies, Heidelberg, Germany) was diluted: 1:200 for western blotting and 1:50 for immunohistofluorescence studies. Rabbit anti-c-Fos (4, Santa Cruz Biotechnologies, Heidelberg, Germany) was diluted 1:200 for western blotting and 1:50 for immunohistofluorescence experiments. Rabbit anti-active Caspase 3 (H-277, Santa Cruz Biotechnologies, Heidelberg, Germany) was diluted 1:200 for western blotting analysis. Rabbit anti-total Caspase 3: (BD Biosciences PharMingen, Franklin Lakes, NJ) was diluted 1:200 for western blotting and immunohistofluorescence experiments.

#### Secondary antibodies

Peroxidase-conjugated AffiniPure Goat anti-Mouse IgG (H^+^L) and peroxidase- conjugated AffiniPure Goat anti-Rabbit IgG (H^+^L; both from Jackson ImmunoResearch, Suffolk, UK) were diluted 1:5000 in PBSTw for secondary detection in western blot analyses. In immunohistofluorescence experiments secondary detection was performed using Alexa Fluor 568-goat anti-mouse IgG (H^+^L; Molecular Probes, Invitrogen, Barcelona, Spain) and Cy3-goat anti-Rabbit IgG (H^+^L; Jackson ImmunoResearch) diluted 1:500 in PBSTx. These antibodies were previously checked for unspecific labeling (4 h incubation onto retinal sections without primary antibody incubation).

## Results

### General overview: number of sequences that are regulated in the retina by optic nerve injury

We performed a time-course analysis of the retinal transcriptome in two different models of pathological RGC death, optic nerve transection and optic nerve crush, and compared them to naïve retinas. Based on anatomic data from our group [3,5], we choose five time points post-lesion (pl), 12 h, 24 h, 48 h, 3 day, 7 day, and 15 day (further details on number and array replicas see Methods). At early time points (from 12 hpl to 3 days post lesion [dpl]) no RGC death is observed. At 7 dpl, 38% (IONT) and 20% (IONC) of the RGCs have died. Finally, 90% and 68% of the RGCs have disappeared two weeks after IONT and IONC, respectively.

**Figure 2:**
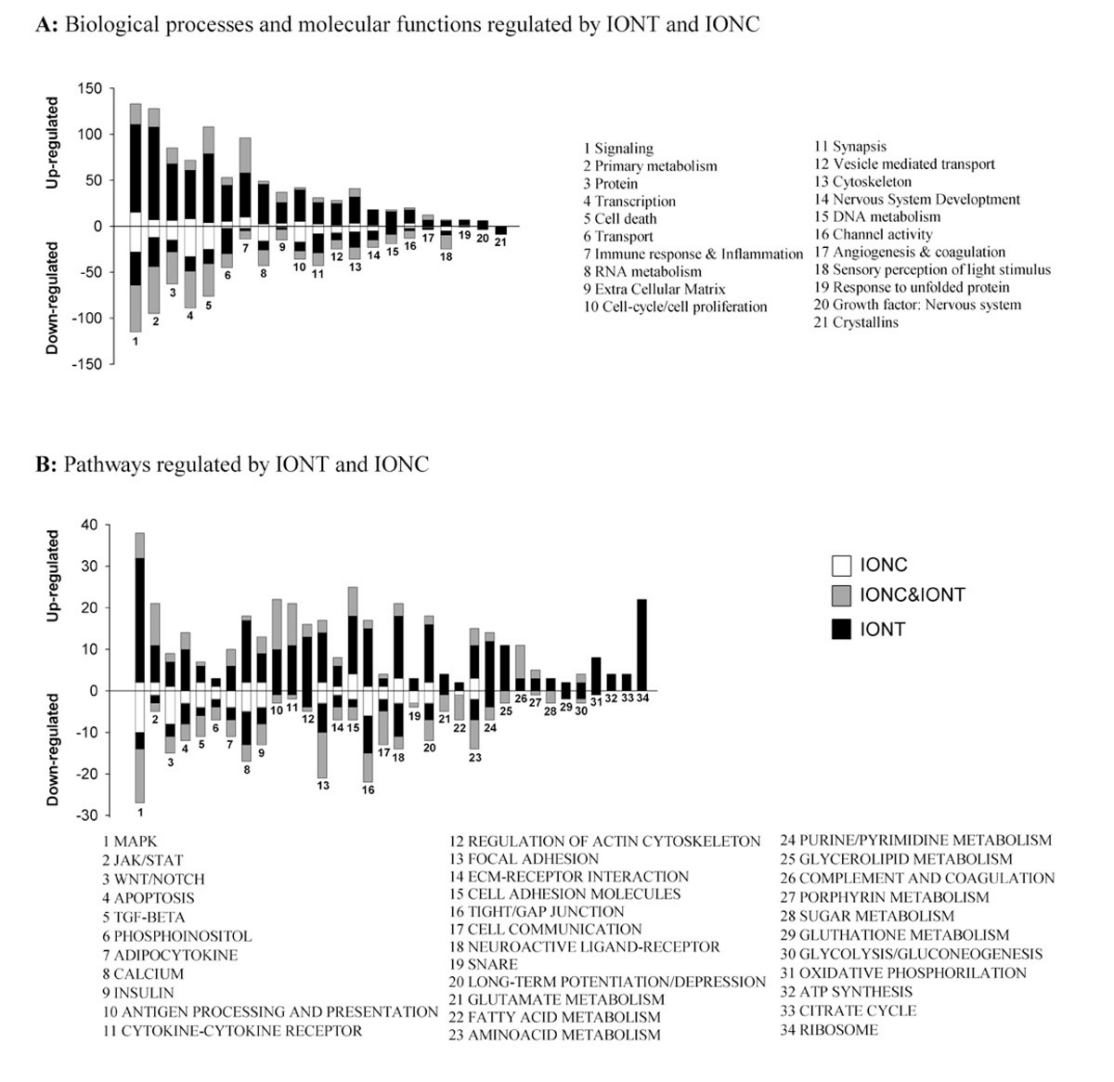
Functional clustering of genes regulated after optic nerve injury in the adult rat retina. **A:** Graph showing which biologic processes and molecular functions are regulated by intraorbital nerve transection (IONT) and intraorbital nerve crush (IONC) in the adult rat retina and the number of up and down-regulated genes in each one. **B:** Graph showing which pathways are regulated by IONT and IONC in the adult rat retina and the number of up- and down-regulated genes in each one. From 1 to 9: pathways related to cell signaling; from 10 to 11 pathways related to inflammation and immune system; from 12 to 16 pathways related to cytoskeleton; from 17 to 21 pathways related to synapse and from 22 to 25, and 27 to 32 pathways related to basic metabolism. Clusters with a pEASE value <1.00E-04 were considered significant. Black bars represent the number of genes of a given cluster specifically regulated by IONT, white bars specifically regulated by IONC and gray bars commonly regulated by both lesions. The number of upregulated genes in each cluster is shown in the positive y-axis and number of downregulated ones in the negative y-axis Abbreviations: IONT: intraorbital nerve transection. IONC: intraorbital nerve crush.

The global number of sequences (genes and ESTs) that were regulated in this study (p value FDR <0,01, B value >0) is shown in Figure 1A. The first observation is that IONT triggers a higher retinal transcriptome regulation (by 36.2%) than IONC does (3,219 sequences regulated after IONT and 1,996 after IONC). This is not due to the extra time point analyzed after IONT (day 15) since if these sequences are removed, IONT regulation is still higher by 29.8% (2,846 IONT sequences regulated from 12 hpl until 7 dpl). Out of all the regulated sequences, 2,071 sequences correspond to genes (Figure 1B), which can be divided into: a) genes regulated only by IONT (1,117, IONT-specific genes); b) genes regulated only by IONC (293, IONC-specific genes); and c) genes regulated after both injuries (661, IONT and IONC common genes).

### Number of sequences regulated after intraorbital nerve transection and intraorbital nerve crush in the adult rat retina and their time course expression

#### Genes

The number of upregulated and downregulated genes and their temporal course of expression are summarized in Figure 1C. Gene regulation is mainly transient after IONC (Figure 1C, right) where 59.6% out of the total IONC-triggered genes are regulated only at one time point, namely at 12 hpl. This differs from IONT where most genes (68.7%) are regulated at more than one time point (Figure 1C, left). Thus, IONT induces mainly a sustained response and IONC a transitory response. In Figure 1C, it is also noticeable that after IONT, 60% of the genes are upregulated whereas after IONC 67% are down-regulated. Genes that are regulated after both lesions follow the same pattern of upregulation or downregulation with just a few exceptions (Appendix 1). However, though similarly regulated after both injuries, they do not always follow the same time course. This is observed in Figure 1C where the gray bars (common genes) in the IONT graph do not correlate to the same time points shown in the IONC graph and vice versa.

**Table 3:** Array validation.

	IONT 12 h		IONT 48 h	
Gene Symbol	qRT–PCR	Array	qRT–PCR	Array
*Ahr*	4.37 (0.78)	9.12 (1.02)	3.85 (0.72)	8.37 (1.40)
*Calr*	*1.09 (0.13)*	*1.11 (1.03)*	1.36 (0.11)	1.83 (1.11)
*Ccl2*	121.15 (33.88)	75.30 (1.22)	86.94 (29.06)	55.59 (1.41)
*Clu*	1.49 (0.12)	1.70 (1.14)	2.17 (0.03)	2.26 (1.02)
*Eef2k*	2.25 (0.34)	1.14 (0.15)	1.69 (0.26)	1.27 (1.13)
*Lcn2*	8.24 (2.28)	16.98 (1.80)	8.52 (0.52)	27.17 (1.12)
*Litaf*	7.09 (0.74)	8.31 (1.15)	6.09 (0.99)	9.14 (1.02)
*Stat1*	3.44 (0.37)	6.52 (1.26)	3.28 (0.82)	7.33 (1.21)
*Tnfrsf12a*	3.64 (0.35)	5.79 (1.09)	3.32 (0.37)	11.76 (1.01)
*Tnfrsf1a*	3.55 (0.02)	7.15 (1.15)	5.63 (0.49)	6.22 (1.11)
				
	IONC 12 h		IONC 48 h	
Gene Symbol	qRT–PCR	Array	qRT–PCR	Array
*Ahr*	3.29 (0.21)	6.65 (1.30)	1.79 (0.41)	1.74 (1.64)
*Calr*	1.62 (0.16)	1.41 (1.35)	1.25 (0.18)	1.71 (1.21)
*Ccl2*	174.77 (117.53)	49.75 (6.16)	55.86 (54.69)	29.54 (3.05)
*Clu*	2.61 (0.53)	2.08 (1.35)	1.54 (0.6)	1.96 (1.04)
*Eef2k*	1.76 (0.28)	2.56 (1.76)	*1.07 (0.28)*	*1.39 (1.13)*
*Lcn2*	11.39 (4.45)	17.09 (3.00)	4.76 (1.76)	16.66 (1.8)
*Litaf*	6.36 (1.2)	6.12 (1.5)	4.65 (1.57)	5.07 (1.27)
*Stat1*	5.69 (2.4)	9.85 (1.9)	3.19 (1.69)	6.30 (1.81)
*Tnfrsf12a*	4.54 (0.78)	4.77 (3.14)	3.78 (1.37)	10.61 (1.50)
*Tnfrsf1a*	4.57 (2.14)	8.88 (1.35)	4.38 (0.95)	5.90 (1.45)

This table shows qRT–PCR and array fold changes of mRNA levels in retinal samples following intraorbital nerve transection (IONT), and intraorbital nerve crush (IONC) at 12 h and 48 h post lesion (hpl). Changes are in relation to naïve retina values that were arbitrarily set to 1. Numbers in italic indicate not significant changes. Standard error of the mean (SEM) for each experiment is shown in brackets. Abbreviations: 12 h: 12 h post-lesion; 48 h: 48 h post-lesion.

#### Expressed sequence tags

The pattern of temporal distribution and regulation of ESTs resembles that of the annotated sequences as shown in Figure 1D. Since the optic nerve injury triggers a high number of gene regulation responses in the retina, we focused this study on the regulation of annotated sequences, leaving aside the ESTs.

### Functional clustering of optic nerve injury-regulated genes

Genes regulated after IONC and IONT were clustered using the web-based tools, DAVID and EASE [24,25]. The main clusters (pEASE <1,00E-04) and cell pathways regulated after IONT and IONC and the number of genes in each one are summarized in Figure 2. A detailed and comprehensive list can be found in Appendix 2 and Appendix 3. Each main cluster was hierarchically divided into sub-clusters based on more specific functions (i.e., main cluster included cell death and sub-clusters included apoptosis, autophagy, and inflammatory response linked to cell death). The highest regulated biologic processes were signaling (cluster 1, pathways 1–9), cytoskeleton and associated processes (cluster 13, pathways 12–16), primary metabolism (clusters 2 and 3 and pathways 22–25 and 27–33 where generation of cellular energy, pathways 30–33, is altered mainly after IONT, although glycolysis and gluconeogenesis are affected by IONC as well), protein metabolism (cluster 3, pathways 21 and 23), immune response and inflammation (cluster 7, pathways 10 and 11, which are mainly upregulated after both injuries), ribosomal protein genes (pathway 34, regulated after IONT but not after IONC), and RNA metabolism, processing, and translation (cluster 8, affected after both lesions but mainly upregulated after IONT and downregulated after IONC).

As expected, cell death was highly regulated (cluster 5 pEASE value 5.60E-08 for IONT and 2.20E-7 for IONC). Our data indicate that the retinal cell death caused by optic nerve injury is apoptotic (pathway 4, pEASE value 2,40E-19 for IONT and 4,80E-06 for IONC) since no other cell death mechanism was found statistically regulated. In total, 186 genes were clustered under cell death out of which 89 and 29 were specific for IONT or IONC, respectively, and 68 were regulated by both lesions (Appendix 3). Some of these genes have been further analyzed.

**Figure 3:**
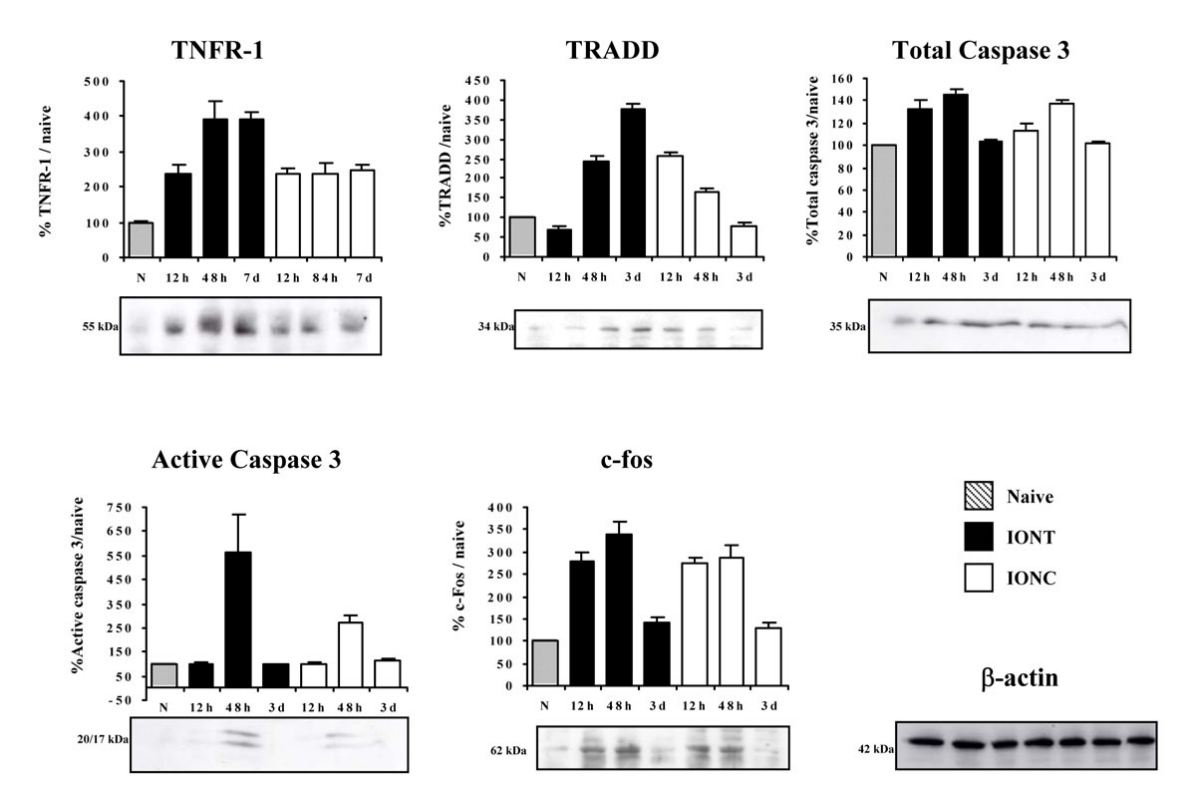
Time course regulation of cell death–related proteins in naïve and optic nerve injured retinas. Western blot time course analyses showing the regulation of tumor necrosis factor receptor superfamily member 1a (TNFR1a), tumor necrosis factor receptor type 1, associated death domain (TRADD), total Caspase 3, active Caspase 3, and c-fos in naïve, intraorbital nerve transection (IONT)-, and intraorbital nerve crush (IONC)-injured retinas. Graphs show quantification of protein signals (n=4 animals per lesion and time point, western blots were replicated three times). The signal from injured retinas is referred to the naïve signal, which was arbitrarily considered 100%. To verify the amount of loaded protein, western blots were incubated with β-actin (an example is shown). Error bars show the standard error of the mean (SEM) for each experiment.

**Figure 4:**
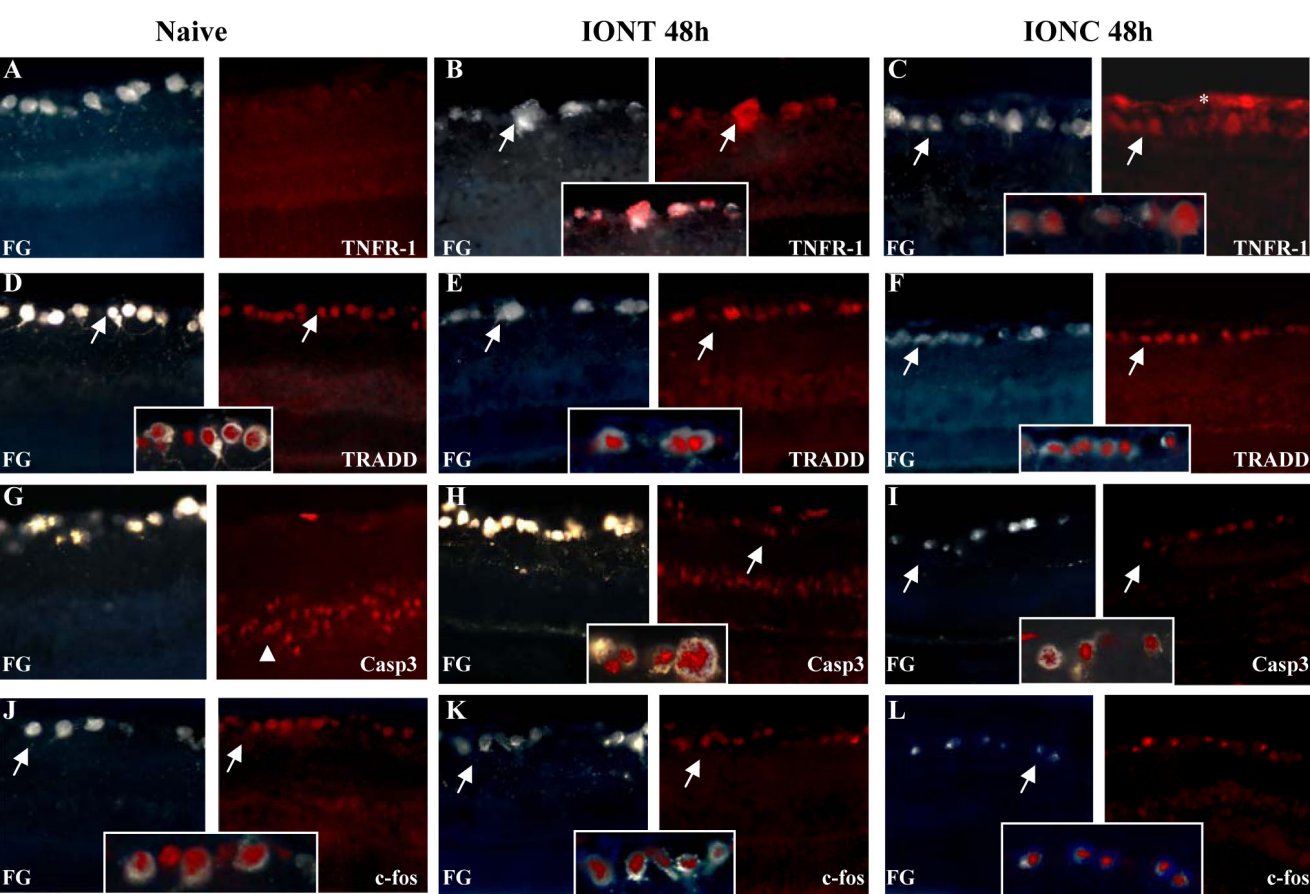
Expression pattern of cell death–related proteins in naïve and optic nerve injured retinas. Immunohistofluorescence analyses for tumor necrosis factor receptor superfamily member 1a (TNFR1a), tumor necrosis factor receptor type 1, associated death domain (TRADD), Caspase 3, and c-fos to fluorogold-traced retinas in naïve, intraorbital nerve transection (IONT) and intraorbital nerve crush (IONC) injured retinas. **A-C:** Expression pattern of TNFR1a (red signal) in naïve retinas (**A**, right), IONT-injured retinas (**B**, right) and IONC-injured retinas (**C**, right). Left images are the corresponding fluorogold (FG) images (blue signal). **D-F:** Expression pattern of TRADD (red signal) in naïve retinas (**D**, right), IONT-injured retinas (**E**, right) and IONC-injured retinas (**F**, right). Left images are the corresponding fluorogold (FG) images (blue signal). **G-I:** Expression pattern of Caspase 3 (red signal) in naïve retinas (**G**, right), IONT-injured retinas (**H**, right) and IONC-injured retinas (**I**, right). Left images are the corresponding fluorogold (FG) images (blue signal). **J-L:** Expression pattern of c-fos (red signal) in naïve retinas (**J**, right), IONT-injured retinas (**K**, right) and IONC-injured retinas (**L**, right). Left images are the corresponding fluorogold (FG) images (blue signal). Magnifications in squares show the co-localization of a given protein with FG-labeled retinal ganglion cells (RGCs). Arrows point to RGC, arrowheads indicate the outer nuclear layer and asterisks mark the nerve layer.

### Array validation

Once genes were annotated and clustered, 10 IONT and IONC upregulated genes related with death and inflammation were chosen for array validation using quantitative-reverse transcriptase-polymerase chain reaction (qRT–PCR). The results are shown in Table 3. The analyzed genes follow the same trend after both experiments. However, differences in net regulation between the results that were obtained after both techniques were observed. These differences can be explained by the variations in experimental set-up and the sensitivity of each technique. In the array experiments, all transcripts hybridize simultaneously and the hybridization is not optimized for each single sequence. In the qRT–PCR, each reaction is optimized for measuring the single cDNA of a transcript that the primers were designed to bind to. For the majority of genes studied using qRT–PCR, their regulation was very similar to that observed in the array experiment. The only exception is *Eef2K* that is regulated after IONT when analyzed by qRT–PCR but not in the array analysis. This was probably due to the low level of upregulation observed in the array experiment 12 h after IONT (1.14 fold change compared to naïve levels). The low level of upregulation together with the variability among the array replicas (SEM: 0.15) resulted in a not significant change (p value FDR=0.07). In conclusion, there is a correlation between both experimental techniques, which validates the array experiment.

### Temporal expression level and retinal pattern distribution of a few death-related proteins

The mRNA of *tumor necrosis factor receptor 1a (TNFR1a), Caspase 3 (Casp3)* and *c-fos* is upregulated after both optic nerve injuries (Appendix 3). We used western blotting to study whether their protein levels followed their mRNA regulation. In addition, we measured active *Casp3* to know whether its upregulation was followed by its activation. Casp3 implication in IONT-induced RGC death has been described previously [24,26,27] but to our knowledge not in the IONC model. In addition, the temporal expression analysis of this executioner protein will provide insight as when the RGCs are committed to death in our models. *TNFR1* has been implicated in RGC death in the mouse retina following optic nerve crush [25]. TNFR1a, depending on its downstream adaptor, could be linked to apoptosis or survival [28]. For this reason, we also measured the regulation of tumor necrosis factor associated death domain (TRADD), which links TNFR1a and TNFR12A (Fn14) signaling to apoptosis [29,30]. Clarify here that the arrays did not contain probes for *TRADD* mRNA and therefore it was not possible to asses for its mRNA regulation. The results are shown in Figure 3, TNFR1a, Casp3, and c-fos proteins are upregulated along time after both injuries. This is in agreement with the array results. c-fos upregulation after both injuries contrasts with other reports [31,32] where this protein does not show regulation. This may be due to the different time points analyzed together with the different sensitivity of the techniques used. The levels of TRADD protein also increased after both injuries in our study. As for the active form of Casp3, it peaked at 48 h after IONT and IONC, which was earlier than previously described [24]. This temporal difference may be a reflection of the distance from the eye where the injury is performed, which is closer in our model. This supports previous reports, which demonstrate that the closer to the eye the injury occurs, the more rapid the RGC degeneration is [5].

To find out whether these upregulated pro-apoptotic proteins were expressed by RGCs, we performed immunohistofluorescence on radial sections from naïve, IONT-, and IONC-injured retinas. To identify RGCs, these were traced with Fluorogold (FG) one week before the injury was performed. Animals were kept 48 h post-optic nerve injury since it was at this time point when the protein levels peaked as shown by the western blot analyses. Results are shown in Figure 4. Naïve and injured RGCs expressed TRADD and c-fos as seen by colocalization of the fluorogold tracer and red fluorescence immunoreactivity. Casp3 and TNFR1a proteins were detected in injured RGCs but not in naïve RGCs.

## Discussion

We have compared the temporal profiling of the retinal transcriptome after two types of optic nerve injury that trigger RGC death, intraorbital nerve transection and intraorbital nerve crush [1-3,5]. The results show that optic nerve injury triggers a plethora of gene regulation responses in the retina as soon as 12 hpl. Injury-regulated sequences fall in three categories, those uniquely regulated after IONT, those uniquely regulated after IONC, and those regulated in a similar way after either of both injuries. There are two different temporal responses, genes that are transiently regulated and genes that have a sustained regulation over time. Three main differences are observed between both injuries, 1) the number of the regulated sequences, 2) the temporal profiles of the regulated sequences, and 3) the majority of upregulation or downregulation. The number of regulated sequences is higher after IONT compared to the number of sequences after IONC. While IONC mainly triggers transient responses that are particularly evident at 12 hpl, IONT mainly triggers a sustained response. Finally, after IONC, the overall response is downregulation whereas after IONT, the main response is upregulation. These differences are likely to reflect the progression of retinal cell death and may account for the different time-courses of RGC death observed after IONT and IONC [2-5].

Fifteen days post-IONT, hundreds of genes are regulated in agreement with previous reports [33]. At this time post-lesion, just a small proportion of RGCs is still alive. Therefore, these changes might be attributable to other retinal cells. However, this does not mean that these cells are dying but rather that they are responding to the lesion inflicted to the RGCs.

Functional clustering of regulated genes revealed that many biologic processes and signaling pathways are involved in the response to optic nerve injury. In agreement with previous studies [34,35] and according to our results, the main cell death mechanism is apoptosis. IONT regulates a higher number of death-related genes than IONC. This concurs with the more rapid course of RGC death observed after IONT than after IONC. We have focused this study on genes directly involved in cell death processes since RGC death is the most pronounced effect after optic nerve injury. There is a possibility that RGC death is not a primary effect of the injuries but rather a secondary one. Thus, it could not be ruled out that the optic nerve injury may primarily affect other processes in the retina such as cell-cell communication or even basic metabolic alterations that will eventually trigger cell death.

**Figure 5:**
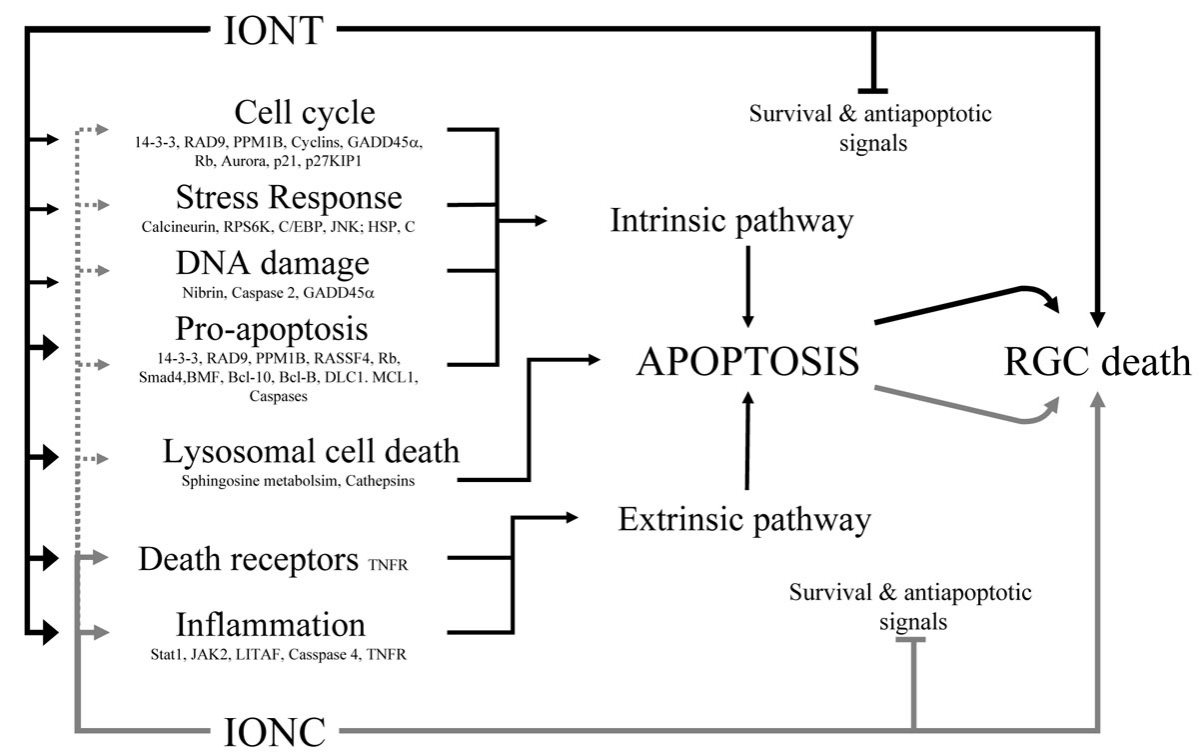
Scheme summarizing the retinal response to intraorbital nerve transection and intraorbital nerve crush leading to cell death. Black lines correspond to intraorbital nerve transection (IONT) and gray ones to intraorbital nerve crush (IONC). Thicker arrowheads symbolize that more genes of a given process are regulated. Dotted lines represent that IONC regulates fewer genes in a process than IONT does. See text for explanation and Appendix 4 for gene interaction and time course regulation.

Since both injuries lead to RGC death, it is likely that the commonly regulated cell death-related genes are responsible for retinal degeneration associated to axonal injury. Therefore, we chose ten of those genes to validate our array data by qRT–PCR. This is the first time that the retinal regulation of these genes by both models of optic nerve injury is reported with the exception of *clusterin*, which has been shown to be upregulated at 15 dpl after IONT [33]. Interestingly, *clusterin, lipocalin 2, litaf*, and *TNFR1a* are also upregulated by other injuries as elevation of intraocular pressure (IOP) or ischemia [36-40]. An increase of Clusterin and Lipocalin 2 expression together with that of ceruloplasmin and early growth response 1 (Egr1) is associated with increased apoptosis and immune-inflammatory responses [41,42]. All of those genes, which were upregulated after IONT and IONC, are also upregulated after ischemia, retinal scraping, experimental glaucoma, and phototoxic lesions (in the case of Egr1). These injuries trigger degeneration of retinal neurons, and so the similar upregulation of these genes after different retinal insults indicates that there might be a common response to injury. Whether such common responses are involved in or may modulate the progression of injury-associated cell death is subject for further studies.

To bring the validation of the arrays one step further and to gain some additional information about the translatability of the transcriptional changes to the protein level, we performed western blot and immunohistofluorescence analyses in naïve, IONT-, and IONC-injured retinas. These results demonstrate that for these genes, protein regulation follows transcriptional regulation. In addition, we showed that these pro-apoptotic proteins are expressed by RGCs, and this result gives support that the identified networks are active in injured RGCs. In conclusion, the expression pattern and regulation of TNFR1a, TRADD, and the executioner protease, Caspase 3, indicate that the extrinsic pathway to apoptosis [43] is active in RGCs undergoing IONT or IONC.

### Signaling maps

IONT- and IONC-regulated genes were loaded into MetaCore^TM^ to search for regulated gene networks and signaling maps. Death signaling maps that were significantly regulated by optic nerve injury (pEASE <1.00E-04) were selected and merged for further analysis and visualization purposes using MapEditor^TM^. The merged map reflects the main gene networks regulated by optic nerve injury that lead to cell death. (Figure 5 and Appendix 4). In addition to the array results, regulation after IONT and IONC for many of the genes shown in the map has been validated by both qRT–PCR and western blot. Besides, some of these proteins have been shown to be expressed by injured RGCs. These maps visualize the retinal responses to IONC and IONT and are based on a significant over-representation of regulated genes. However, we are aware that these are partial maps of the retinal response to optic nerve injury since just 47 out of the 189 death-related genes which are regulated have been included. Furthermore, the data that support the maps belong to “known gene interactions.” Therefore, these maps are not irrefutable and should be considered as a step to unravel the molecular mechanisms underlying optic nerve injury- induced retinal degeneration. As shown in Figure 5 and in detail in the Appendix 4, eight main cellular processes, which may lead to cell death, were identified as being involved in the retinal response to optic nerve injury: stress response, induction of apoptosis by extracellular signals (extrinsic pathway), by intracellular signals (pro-apoptotic genes, intrinsic pathway), regulation of survival signals, activation of immune-inflammatory pathways, DNA damage, cell cycle deregulation, and the lysosomal death pathway. Interestingly, both IONT and IONC trigger TNFR1-TRADD signaling, which leads to the activation of the extrinsic apoptosis pathway [29,30,43,44]. This is strengthened by the upregulation after IONT and IONC of *caspases 3* and *11* (*caspase 4* in humans) and of another member of the tumor necrosis factor family, *TNRSF12A* (Fn14), which is also linked to apoptosis through *TRADD* signaling [29,30,45]. This is further supported by the upregulation of caspase 8 in axotomized RGCs reported by Weishaupt et al., 2003. [46]. Pro-apoptotic genes, associated to the intrinsic apoptotic pathway, are regulated after IONT and IONC (*14–3–3, RAD9, RASSF4, Rb protein, Smad4, PPM1B*), although IONT (*BMF, Bcl.10, Bax, Bcl-B, DLC1, MCL1*) seems to trigger a higher response than IONC does. Some of these genes are linked to cell cycle control as *14–3–3, RAD9*, and *PPM1B*. Cell cycle deregulation has been reported as a threshold for cell death [47], and genes involved in the cell cycle control are regulated after both injuries (*Cyclin G1, GADD45α, Rb protein*, and aforementioned). Again, there is a higher response after IONT (*Cyclins, CDK1, Aurora, p27KIP1, p21*) compared to IONC. Interestingly, an upregulation of cell cycle-related genes is observed as well after spinal cord lesion, another model of central nervous system axonal injury and neuronal degeneration [48]. DNA damage is another trigger of apoptosis [49] and *GADD45α* and *nibrin,* involved in this process, are regulated after optic nerve injury (the former after both lesions and the latter only after IONT). Increased levels of caspase 2 are important in cell death activated by DNA damage [50], and we found this gene to be downregulated by both injuries, which suggests that DNA damage is not the main pathway for the optic nerve injury-induced retinal degeneration. Survival and anti-apoptotic signals [51] such as *XIAP, ERK2, SOS, c-Raf-1*, and *YY1* are downregulated. It is worth highlighting here that among the genes linked to apoptosis triggered by mitochondrial proteins (Bad and Bid among others) only *Bax* is regulated. Interestingly, it is regulated only by transection and quite late after the injury at 3 dpl. This would agree with previous reports where administration of Bcl-2 simultaneously to IONT did not delay RGC-associated death [6]. Stress response to injury is evidenced by the regulation of *calcineurin* and *RPS6K* after IONT and *C/EBP*, *JNK*, and *heat shock protein* [52,53] genes after IONT and IONC. There is a strong immune-inflammatory response [54] as seen by the upregulation of *JAK2, Litaf, Stat 1*, and *caspase 11* among others. It is important to highlight here that some of these genes such as caspase 11, stat 1, and litaf are also related to the apoptotic pathway [55-57]. Finally, optic nerve injury regulates several *cathepsins*, (*H, L*, and S after IONT and *C* and *Z* after IONT and IONC). Cathepsins are proteases located in the lysosomes, which have a role in apoptosis through the “lysosomal pathway of apoptosis” [57-59]. Lysosomal permeabilization and release of cathepsins are facilitated by sphingosine, a lipid whose overexpression is linked to apoptosis [60]. Our array study reveals that the RNA of some enzymes linked to sphingosine metabolism are soon upregulated after IONT (*Sphk1* and *Sgpl1*) and after IONC (*Sphk1* and *2*) [58-60]. Cathepsins’ role in apoptosis has two sides. First, they act as proteases cleaving and thus activating caspases. Second, they act on the mitochondria to induce mitochondrial dysfunction [58,61].

Together, all this indicates that IONT triggers a more acute and more pronounced apoptotic response than IONC [62]. First, more genes are regulated after IONT, and those, which are commonly regulated, show in general a longer lasting regulation after transection than after crush. Second, the regulation of the extrinsic apoptotic pathway and immune-inflammatory response is similar after IONT and IONC. However, lysosomal cell death, DNA damage, cell cycle control, and pro-apoptotic signals that may activate the intrinsic apoptotic pathway are mostly deregulated after IONT. All these may account for the slower degeneration of RGC after optic nerve crush than after transection and, most importantly, it highlights the common responses that may cause the RGC degeneration triggered by optic nerve injury.

## Appendix 1. Genes commonly regulated by intraorbital nerve crush and intraorbital nerve transection that follow opposite regulation

To access the data, click or select the words “Appendix 1.” This will initiate the download of a compressed (pdf) archive that contains the file.

The data shown here are statistically significant fold changes in gene expression (p value false discovery rate (FDR) limma and maSigpro is <0.01 and B value is >0). Values below 1 mean downregulation, and above 1 upregulation compared to naïve samples. Empty cells refer either to control level of expression, which is 1, or to not statistically significant changes. Abbreviations: IONT intraorbital nerve transection; IONC intraorbital nerve crush, h: hours, d: days.

## Appendix 2. Functional clusters regulated in the retina after intraorbital nerve transection and intraorbital nerve crush.

To access the data, click or select the words “Appendix 2.” This will initiate the download of a compressed (pdf) archive that contains the file

Genes were clustered using DAVID and PubMed reports. Clusters with a pEASE value <1,00E-04 were considered significant. The main biologic processes are shown in red, and their sub-clusters are shown in black. Abbreviations: IONT represents intraorbital nerve transection; IONC represents intraorbital nerve crush; FDR represents false discovery rate.

## Appendix 3. Time course gene regulation in adult rat retinas after intraorbital nerve transection and intraorbital nerve crush and functional clustering

To access the data, click or select the words “Appendix 3.” This will initiate the download of a compressed (pdf) archive that contains the file.

The data shown here are statistically significant fold changes in gene expression (p value false discovery rate (FDR) limma and maSigpro is <0.01 and B value is >0). Values below 1 mean downregulation, and above 1 upregulation compared to naïve samples. Empty cells refer either to control level of expression, which is 1, or to not statistically significant changes. Genes were clustered using DAVID [22] and PubMed reports. Clusters with a pEASE value of less than 1.00E-04 were considered significant. As population manager, backgrounds of Affymetrix RAE230.2 and *Rattus norvegicus* genome were used. To avoid repetitions, each gene was ascribed to one biologic process. The main biologic processes are shown in red bold font and their sub-clusters are shown in black bold font.

## Appendix 4. Cell-death signaling maps based on retinal gene regulation after intraorbital nerve crush and intraorbital nerve transection.

To access the data, click or select the words “Appendix 4.” This will initiate the download of a compressed (pdf) archive that contains the file

**A:** Cell-death signaling map summarizing the regulation and gene interactions of retinal-regulated genes after intraorbital nerve crush (IONC) and intraorbital nerve transection (IONT) injuries. Data from our array analyses were loaded in MetacoreTM and MapEditorTM softwares (Genego Inc.). Significantly regulated MetacoreTM cell death signaling maps were merged and a custom map was generated using curated gene interactions. Gene interactions are represented by directional color-coded arrows: gray unknown, green: activation, red: repression or inactivation of the target. Interactions are as follow: B: binding; +P: phosphorylation; TR: transcription; C: cleavage; IE: influence on expression; ?: not specified. TNFR1a, TRADD, c-fos (AP-1 complex) and Caspase 3 protein expression has been analyzed by western blotting, and Stat-1, TNFR1a, and TNFR12a mRNA regulation has been validated by qRT–PCR. **B:** Temporal expression of IONT- and IONC-regulated genes shown in the signaling map in **A**. Data shown here are significant fold changes. Values below 1 mean downregulation and above 1 upregulation compared to naïve samples.
